# Determinants of loss to care and risk of clinical progression in PLWH who are re-engaged in care after a temporary loss

**DOI:** 10.1038/s41598-021-88367-5

**Published:** 2021-05-05

**Authors:** Cristina Mussini, Patrizia Lorenzini, Alessandro Cozzi-Lepri, Alessia Mammone, Giovanni Guaraldi, Giulia Marchetti, Miriam Lichtner, Giuseppe Lapadula, Sergio Lo Caputo, Andrea Antinori, Antonella d’Arminio Monforte, Enrico Girardi

**Affiliations:** 1 Infectious Diseases Clinic, University of Modena and Reggio Emilia, Modena, Italy; 2 National Institute for Infectious Diseases ‘L. Spallanzani’, Via Portuense 292, 00149 Rome, Italy; 3 Institute for Global Health, UCL, London, UK; 4 Clinic of Infectious Diseases, San Paolo Hospital, University of Milan, Milan, Italy; 5 Department of Public Health and Infectious Diseases Unit, Sapienza University of Rome, Polo Pontino, Latina, Italy; 6 Division of Infectious Diseases, San Gerardo Hospital, Monza, Italy; 7 Clinic of Infectious Diseases, University of Foggia, Foggia, Italy

**Keywords:** Health care, Medical research, Risk factors

## Abstract

The risk of developing AIDS is elevated not only among those with a late HIV diagnosis but also among those lost to care (LTC). The aims were to address the risk of becoming LTC and of clinical progression in LTC patients who re-enter care. Patients were defined as LTC if they had no visit for ≥ 18 months. Of these, persons with subsequent visits were defined as re-engaged in care (RIC). Factors associated with becoming LTC and RIC were investigated. The risk of disease progression was estimated by comparing RIC with patients continuously followed. Over 11,285 individuals included, 3962 became LTC, and of these, 1062 were RIC. Older age, presentation with AIDS and with higher HIV-RNA were associated with a reduced risk of LTC. In contrast, lower education level, irregular job, being an immigrant and injecting-drug user were associated with an increased LTC probability. Moreover, RIC with HIV-RNA > 200 copies/mL at the re-entry had a higher risk of clinical progression, while those with HIV-RNA ≤ 200 copies/mL had a higher risk of only non-AIDS progression. Patients re-entering care after being LTC appeared to be at higher risk of clinical progression than those continuously in care. Active strategies for re-engagement in care should be promoted.

## Introduction

Over the last 30 years, great advances have been achieved in HIV care. However, even in resource-rich countries, symptomatic AIDS has not disappeared, and many people with advanced HIV disease are admitted to the hospital every year and die. AIDS is the most common cause of death in people living with HIV (PLWH) in the UK, and mortality remains higher in these individuals than in the general population^1^. In Italy, in 2019, 1306 out of 2224 new diagnoses that included reported CD4 counts had a value < 350 cells/µL (58.7%), and 39.7% had a value < 200 cells/µL^2^. The Joint United Nations Program on HIV/AIDS launched an agenda to achieve the elimination of AIDS, introducing the “90–90–90 targets”, the so-called treatment cascade. This public health campaign aims to achieve three ambitious goals by 2020: HIV diagnosis in 90% of all PLWH, the provision of antiretroviral therapy (ART) to 90% of the diagnosed individuals, and the achievement of viral suppression in 90% of the treated patients. The second 90% target, that is, retention in care, is a critical step in the management of PLWH and is associated with improved survival, decreased HIV-related complications, and reduced HIV transmission^3–5^. In addition to the fact that retention in care is associated with improved HIV disease-specific outcomes, it is also the step in the HIV care continuum in which the largest proportion of dropouts is observed^6^.

Altogether, these data suggest that progression to advanced HIV disease could be observed not only among those with a late diagnosis of HIV infection but also among those diagnosed early in the course of the infection who are subsequently lost to care; the latter account for up to 62% of all AIDS cases^7^. Risk factors for progression to AIDS in patients who were lost to care have been identified, including psychiatric comorbidities; social issues, such as being immigrants; and alcohol and substance abuse^8^.

Retention in care is a dynamic process, and the treatment cascade is not unidirectional towards loss to follow-up, as a non-negligible proportion of PLWH may be re-engaged in care at different steps of the cascade. Temporary versus permanent loss to care may identify a time-dependent definition of “gap in care” or “lost to care”.

Risk factors associated with a temporary loss to care include younger age, crack/cocaine use, food insecurity, financial and housing instability and phone number changes in the past year, limiting the possibility of re-engaging these individuals^9^.

The ICONA Foundation Study cohort (ICONA) is the largest HIV cohort in Italy and historically has been able to track some key steps of the continuum of care of PLWH, offering a nationally representative picture of HIV care.

The primary aim of this analysis was to describe the risk factors for temporary and permanent loss to care in PLWH enrolled in the ICONA Foundation Study cohort. The secondary aim of the study was to estimate the risk of clinical progression by comparing PLWH retained in care with PLWH who experienced a temporary loss to care and then re-engaged in care.

## Results

**Figure 1:**
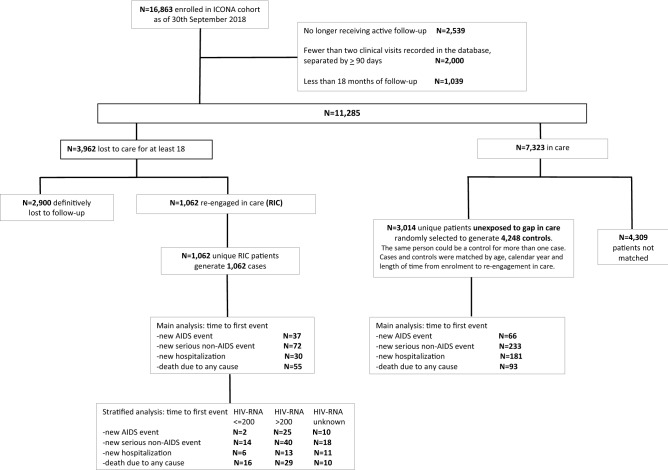
Flowchart of the patients’ disposition from cohort enrolment to the analysis endpoints.

Out of 16,863 patients enrolled in the Icona Foundation Study cohort over the period January 1997–March 2017, 11,285 (67%) who satisfied the inclusion criteria were included in this analysis (Fig. 1). Overall, 77% were males, and 83% were of Italian origin, with a median age at enrolment of 37 years (Interquartile range, IQR 31–45) (Table 1a). Forty-two percent of the patients were stably employed, 14% were self-employed and 14% were unemployed. In approximately 30% of the study population, the highest level of education achieved was secondary school or lower, while 29% had completed college and 10% had a university degree.

**Table 1:** Main characteristics at the enrolment of the study population (a) and of the RIC patients (b) compared with LTC not returning to the care (c).

	(a) Overall study population N = 11,285	(b) RIC N = 1062	(c) LTC no RIC N = 2900	p-value
**Gender**				
M	8724 (77.3%)	734 (69.1%)	2124 (73.2%)	0.011
F	2561 (22.7%)	328 (30.9%)	776 (26.8%)	
**Age**				
18–35	4877 (43.2%)	548 (51.6%)	1484 (51.2%)	0.201
36–50	4911 (43.5%)	441 (41.5%)	1163 (40.1%)	
> 50	1497 (13.3%)	73 (6.9%)	253 (8.7%)	
**Mode of HIV acquisition**				
Heterosexual	4309 (38.2%)	409 (38.5%)	1061 (36.6%)	< 0.001
PWID	1696 (15.0%)	330 (31.1%)	568 (19.6%)	
MSM	4486 (39.8%)	269 (25.3%)	1075 (37.1%)	
Other/unknown	794 (7.0%)	54 (5.1%)	196 (6.7%)	
**Job**				
Employed	4746 (42.1%)	459 (43.2%)	1106 (38.1%)	< 0.001
Unemployed	1566 (13.9%)	223 (21.0%)	559 (19.3%)	
Self-employed	1618 (14.3%)	142 (13.4%)	404 (13.9%)	
Occasional	405 (3.6%)	56 (5.3%)	162 (5.6%)	
Student	327 (2.9%)	23 (2.1%)	108 (3.7%)	
Retired/invalid/housewife	701 (6.2%)	89 (8.4%)	202 (7.0%)	
Other	294 (2.6%)	19 (1.8%)	66 (2.3%)	
Unknown	1628 (14.4%)	51 (4.8%)	293 (10.1%)	
**Education level**				
Primary school	758 (6.7%)	124 (11.7%)	262 (9.0%)	< 0.001
Middle school	2558 (22.7%)	332 (31.3%)	637 (22.0%)	
High school/university	4484 (39.7%)	338 (31.8%)	1114 (38.4%)	
Unknown	3485 (30.9%)	268 (25.2%)	887 (30.6%)	
**Nationality**				
Italian	9338 (82.8%)	896 (84.4%)	2286 (78.8%)	< 0.001
Other	1947 (17.2%)	166 (15.6%)	614 (21.2%)	
**Presentation with AIDS**				
No	10,145 (89.9%)	973 (91.6%)	2654 (91.5%)	0.910
Yes	1139 (10.1%)	89 (8.4%)	246 (8.5%)	
**HCV co-infection**				
No	7626 (67.5%)	575 (54.1%)	1912 (65.9%)	< 0.001
Yes	1769 (15.7%)	342 (32.2%)	573 (19.8%)	
Unknown	1890 (16.8%)	145 (13.7%)	415 (14.3%)	
**CD4 count, cells/µL**				
0–200	2708 (24.0%)	214 (20.2%)	617 (21.3%)	0.038
201–350	2020 (17.9%)	158 (14.9%)	529 (18.2%)	
351–500	2409 (21.4%)	232 (21.9%)	638 (22.0%)	
> 500	3842 (34.0%)	438 (41.2%)	1063 (36.7%)	
Unknown	306 (2.7%)	20 (1.8%)	53 (1.8%)	
**HIV RNA, copies/mL**				
< 500,000	9528 (84.4%)	926 (87.2%)	2547 (87.8%)	0.014
≥ 500,000	1032 (9.2%)	60 (5.6%)	206 (7.1%)	
Unknown	725 (6.4%)	76 (7.2%)	147 (5.1%)	

Out of the 11,285 included patients, 3962 (35%) became LTC during follow-up, including 1062 (26.8%) participants who became RIC by re-entering the cohort after a gap in care and 2900 (73.2%) who remained lost to care at the time of this analysis (Table 1b,c). RIC were more frequently female, Italian and employed, they showed a higher proportion of PWID and of HCV co-infected, they showed better virological and immunological parameters at enrolment than LTC patients not returning to the care.

**Figure 2:**
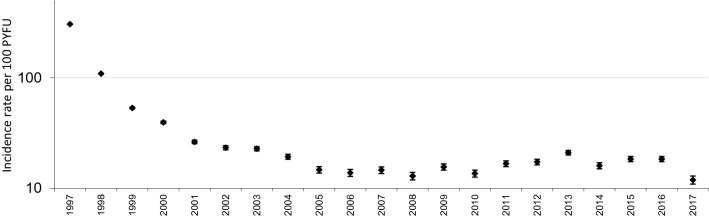
Incidence rate of being LTC according to the calendar year.

The median time from the date of enrolment in the study to becoming LTC was 13.6 years (95% confidence interval, CI 13.0–14.3). The yearly incidence rate of becoming LTC decreased from 1997 to 2005, from 306 per 100 PYFU (95% CI 176–526) to 14.7 (12.2–17.9), with no major changes over the subsequent 10 years, ranging from 13.8 (95% CI 11.4–16.8) per 100 PYFU in 2006 to 18.4 (17.2–19.6) in 2016 (Fig. 2) (the sample size was too small to provide a precise estimate for 2017).

In multivariable analyses, older age, presentation with AIDS and worse virological and immunological conditions at enrolment were independently associated with a reduced risk of becoming LTC (Table 2a). In contrast, a lower level of education, having an irregular job, being an immigrant and contracting HIV through injecting drugs were factors associated with a higher risk of becoming LTC (Table 2a). No association was found with alcohol and/or drug abuse. The results were similar in a sensitivity analysis in which patients who became RIC were not counted as events (Table 2b). In a separate analysis evaluating the association with HIV-RNA as a time-dependent factor, a current HIV-RNA load ≤ 200 copies/mL was found to be associated with a reduced risk of becoming LTC (adjusted Hazard Ratio, HR 0.67, 95% CI 0.62–0.73, vs. current HIV-RNA load > 200 copies/mL).

**Table 2:** Factors associated with time to LTC (a) including and (b) excluding RIC by means of Cox regression.

	(a) Outcome: time to LTC				(b) Sensitivity analysis Outcome: time to LTC (not including RIC patients)			
	Adj HR	95% CI		p-value	Adj HR	95% CI		p-value
**Gender**								
M	1.00				1.00			
F	0.94	0.86	1.03	0.201	0.93	0.83	1.03	0.169
**Age**								
18–35	1.00				1.00			
36–50	0.85	0.79	0.91	< 0.001	0.84	0.77	0.91	< 0.001
> 50	0.70	0.62	0.80	< 0.001	0.69	0.60	0.80	< 0.001
**Mode of HIV acquisition**								
Heterosexual	1.00				1.00			
PWID	1.32	1.17	1.50	< 0.001	1.36	1.17	1.58	< 0.001
MSM	1.04	0.95	1.14	0.419	1.08	0.97	1.20	0.140
Other/unknown	1.06	0.92	1.22	0.405	1.14	0.97	1.34	0.100
**Job**								
Employed	1.00				1.00			
Unemployed	1.45	1.32	1.59	< 0.001	1.51	1.35	1.69	< 0.001
Self-employed	1.05	0.95	1.16	0.372	1.09	0.97	1.23	0.137
Occasional	1.16	1.00	1.35	0.052	1.16	0.97	1.38	0.102
Student	1.32	1.10	1.59	0.003	1.43	1.17	1.76	0.001
Retired/invalid/housewife	1.07	0.93	1.24	0.337	1.11	0.94	1.32	0.227
Other	0.96	0.76	1.21	0.736	0.93	0.72	1.22	0.617
Unknown	0.91	0.79	1.04	0.172	0.93	0.80	1.08	0.341
**Education level**								
Primary school	1.22	1.08	1.38	0.002	1.21	1.04	1.41	0.012
Middle school	0.97	0.89	1.06	0.489	0.95	0.86	1.06	0.370
High school/university	1.00				1.00			
Unknown	1.41	1.29	1.55	< 0.001	1.39	1.25	1.54	< 0.001
**Nationality**								
Italian	1.00				1.00			
Other	1.73	1.58	1.90	< 0.001	1.79	1.61	1.98	< 0.001
**Presentation with AIDS**								
No	1.00				1.00			
Yes	0.83	0.73	0.94	0.005	0.84	0.72	0.98	0.023
**HCV co-infection**								
No	1.00				1.08	0.93	1.24	0.319
Yes	1.12	0.99	1.26	0.064	1.00	0.90	1.12	0.956
Unknown	1.02	0.93	1.13	0.661	0.95	0.85	1.07	0.435
**CD4 count, cells/µL**								
0–200	0.91	0.83	1.01	0.075	0.95	0.85	1.07	0.435
201–350	0.90	0.82	0.99	0.028	0.95	0.85	1.06	0.327
351–500	0.93	0.86	1.01	0.104	0.94	0.85	1.04	0.202
> 500	1.00				1.00			
Unknown	0.86	0.66	1.11	0.248	0.85	0.62	1.16	0.292
**HIV-RNA, copies/mL**								
< 500,000	1.00				1.00			
≥ 500,000	0.84	0.74	0.96	0.013	0.85	0.73	0.99	0.032
Unknown	1.02	0.88	1.20	0.763	1.04	0.86	1.26	0.669

*Adj HR* adjusted hazard ratio

Among the 3963 PLWH who met the LTC definition, 1,062 patients (26.8%) were defined as becoming RIC when they re-entered the cohort after a mean period of 2.7 years (SD  1.7, min-max 1–17.3 years). Seven hundred eighty-seven patients out of these 1062 RIC (74.1%) had HIV-RNA loads available within 7 days after re-engagement, and 356/787 (42.2%) had HIV-RNA loads ≤ 200 copies/mL and 431 (54.8%) HIV-RNA > 200 copies/mL. Table 3 describes the main characteristics of the RIC population according to their HIV-RNA level (≤ 200 vs. > 200 copies/mL) at the point of re-entry into care. Individuals who were RIC and had HIV-RNA loads ≤ 200 copies/mL were more likely to be PWID and HCV-Ab-positive, with a lower level of education and with a higher CD4 count at enrolment respect to those with HIV-RNA > 200 copies/mL.

**Table 3:** Comparison of main characteristics at the enrolment between RIC patients according to the level of HIV-RNA at the re-entry.

	RIC with HIV-RNA ≤ 200 copies/ml at re-entry	RIC with HIV-RNA > 200 copies/ml at re-entry	p-value
	n = 356	n = 431	
**Gender**			
M	256 (71.9%)	284 (65.9%)	0.070
F	100 (28.1%)	147 (34.1%)	
**Age**			
18–35	165 (46.4%)	243 (56.4%)	< 0.001
36–50	152 (42.7%)	175 (40.6%)	
> 50	39 (11.0%)	13 (3.0%)	
**Mode of HIV acquisition**			
Heterosexual	154 (43.3%)	148 (34.3%)	< 0.001
PWID	81 (22.8%)	160 (37.1%)	
MSM	96 (27.0%)	104 (24.1%)	
Other/unknown	25 (7.0%)	19 (4.4%)	
**Job**			
Employed	159 (44.7%)	186 (43.2%)	0.058
Unemployed	65 (18.3%)	99 (23.0%)	
Self-employed	42 (11.8%)	64 (14.9%)	
Occasional	19 (5.3%)	20 (4.6%)	
Student	6 (1.7%)	13 (3.0%)	
Retired/invalid/housewife	32 (9.0%)	30 (7.0%)	
Other	10 (2.8%)	4 (0.9%)	
Unknown	23 (6.5%)	15 (3.5%)	
**Education level**			
Primary school	40 (11.2%)	48 (11.1%)	0.020
Middle school	95 (26.7%)	157 (36.4%)	
High school/university	131 (36.8%)	124 (28.8%)	
Unknown	90 (25.3%)	102 (23.7%)	
**Nationality**			
Italian	289 (81.2%)	370 (85.8%)	0.077
Other	67 (18.8%)	61 (14.2%)	
**Presentation with AIDS**			
No	327 (91.9%)	392 (90.9%)	0.654
Yes	29 (8.1%)	39 (9.1%)	
**HCV co-infection**			
No	222 (62.4%)	209 (48.5%)	< 0.001
Yes	82 (23.0%)	164 (38.0%)	
Unknown	52 (14.6%)	58 (13.5%)	
**CD4 count, cells/µL**			
0–200	88 (24.7%)	73 (16.9%)	< 0.001
201–350	54 (15.2%)	60 (13.9%)	
351–500	86 (24.2%)	77 (17.9%)	
> 500	120 (33.7%)	216 (50.1%)	
Unknown	8 (2.2%)	5 (1.2%)	
**HIV RNA, copies/mL**			
< 500,000	301 (84.6%)	384 (89.1%)	0.167
≥ 500,000	25 (7.0%)	21 (4.9%)	
Unknown	30 (8.4%)	26 (6.0%)	

We have also identified factors associated with the probability of re-entering care with HIV-RNA loads > 200 copies/mL among the LTC population. In this analysis, younger age, a higher CD4 count, female sex, a lower level of education, and HCV co-infection were independently associated with a higher chance of achieving the outcome (Table 4).

**Table 4:** Factors associated with re-engagement in care with HIV-RNA > 200 copies/mL vs LTC no RIC by means of logistic regression.

	Logistic regression Outcome: re-engagement in care with HIV-RNA loads > 200 copies/mL			
	Adj OR	95% CI		p-value
**Gender**				
M	1.00			
F	1.34	1.01	1.78	0.040
**Age**				
18–35	2.15	1.17	3.95	0.013
36–50	2.08	1.13	3.81	0.018
> 50	1.00			
**Mode of HIV acquisition**				
Heterosexual	1.00			
PWID	1.05	0.72	1.54	0.782
MSM	1.00	0.71	1.39	0.985
Other/unknown	0.82	0.49	1.40	0.476
**Job**				
Employed	1.00			
Unemployed	0.94	0.71	1.26	0.699
Self-employed	1.04	0.75	1.43	0.831
Occasional	0.75	0.44	1.25	0.269
Student	0.97	0.51	1.83	0.923
Retired/invalid/housewife	0.73	0.46	1.16	0.183
Other	0.54	0.19	1.58	0.262
Unknown	0.71	0.39	1.30	0.266
**Education level**				
Primary school	1.55	1.04	2.31	0.032
Middle school	1.46	1.10	1.94	0.009
High school/university	1.00			
Unknown	1.07	0.79	1.46	0.653
**Nationality**				
Italian	1.00			
Other	0.97	0.70	1.36	0.874
**Presentation with AIDS**				
No	1.00			
Yes	1.35	0.88	2.06	0.166
**HCV co-infection**				
No	1.00			
Yes	1.43	1.00	2.05	0.048
Unknown	1.20	0.86	1.68	0.283
**CD4 count, cells/µL**				
0–200	1.00			
201–350	1.08	0.72	1.61	0.720
351–500	1.09	0.74	1.62	0.653
> 500	1.77	1.25	2.50	0.001
Unknown	0.99	0.34	2.86	0.982
**HIV RNA, copies/mL**				
< 500,000	1.00			
≥ 500,000	0.86	0.52	1.42	0.557
Unknown	1.39	0.85	2.27	0.188

*Adj OR* adjusted odds ratio

The median change in CD4 cell counts in the RIC population with HIV-RNA loads > 200 copies/mL was − 128 cells/µL (IQR − 287, − 20), and as expected, a longer duration of the gap in care was associated with a larger decrease in the CD4 cell count (Table 5). In contrast, the median change in CD4 cell count in the RIC population with HIV-RNA loads ≤ 200 copies/mL at the time of re-entry was + 62 cell/mmc (IQR − 65, + 202).

**Table 5:** Median value and interquartile range (IQR) of CD4 cell count before and after the gap in care according to gap duration and to HIV-RNA level at re-entry (a) HIV-RNA > 200 copies/mL and (b) HIV-RNA ≤ 200 copies/mL.

(a)	Gap in care duration, months	N of subjects with CD4 count available before and after the gap	Last CD4 before the gap, cells/μL, median (IQR)	CD4 at re-engage, cells/μL, median (IQR)	Change of CD4, cells/μL, median (IQR)	p-value
RIC with HIV-RNA > 200 copies/mL at re-entry	18–24	175	527 (322–695)	395 (180–624)	− 93 (− 230; + 2)	< 0.001
	24–36	139	495 (335–657)	332 (132–489)	− 161 (− 287; − 17)	< 0.001
	> 36	102	568 (342–777)	283 (157–526)	− 196 (− 406; − 74)	< 0.001
	Total	416	528 (335–699)	349 (157–543)	− 128 (− 287; − 20)	< 0.001
**(b)**						
RIC with HIV-RNA ≤ 200 copies/mL at re-entry	18–24	202	640 (441–853)	692 (503–906)	+55 (− 68; + 179)	0.003
	24–36	89	480 (373–768)	625 (452–817)	+62 (− 56; + 184)	0.006
	> 36	75	524 (365–663)	651 (408–934)	+103 (− 62; + 358)	0.009
	Total	342	571 (410–813)	663 (461–896)	+ 62 (− 65; + 202)	< 0.001

### HIV disease progression

Patients who were RIC (n = 1062) were matched with 4248 controls. 4248 controls were randomly selected from 3014 unique patients to achieve a 1:4 exposed/unexposed ratio. The same unexposed control participants could be matched to one or more patients in the RIC population. Over 27,272 person-years of follow-up (PYFU) after the date of re-entry into care, a total of 767 clinical events occurred (103 [13.4%] AIDS events, 305 [39.8%] serious non-AIDS events, 211 [27.5%] hospitalizations, 148 [19.3%] deaths, for an overall incidence rate of 2.8 per 100 PYFU (95% CI 2.6–3.0).

In the first year after re-engagement in care, among 1062 patients who became RIC, 25 patients experienced a new AIDS event [8 *Pneumocystis jirovecii* pneumonia (PcP), 4 lymphoma, 4 wasting syndrome, 2 oesophageal candidiasis, 2 bacterial pneumonia, 2 pulmonary tuberculosis (TB), 1 cryptosporidiosis, 1 Mycobacteriosis other than tuberculosis (MOTT), 1 cerebral toxoplasmosis], 7 patients experienced two concomitant new AIDS events (1 oesophageal candidiasis + PcP, 1 bacterial pneumonia + Kaposi sarcoma, 1 pulmonary TB + extrapulmonary TB, 1 cryptosporidiosis + cytomegalovirosis, 2 cytomegalovirosis + PcP, 1 Kaposi sarcoma + MOTT) and 14 experienced a new serious non-AIDS event (9 cancer, 3 chronic renal impairment, 2 myocardial infarction).

After adjusting for the set of chosen confounders (see “Methods” section/footnote of Table 6), RIC status was associated with a significantly higher risk of clinical progression compared to retention in care (Table 6a). Of note, the association was stronger after restricting the analysis to the subset of patients who were RIC who had HIV-RNA loads > 200 copies/mL at the time of re-entering care or patients with unknown HIV-RNA (Table 6a). Similar results were found when performing a sensitivity analysis in which clinical events that occurred within 3 months of the date of re-entry into care were not counted as events (Table 6b). A second sensitivity analysis using a definition of LTC of 12 months also showed similar results (Supplementary Table S1).

**Table 6:** Crude and adjusted Hazard Ratio (adj HR) and relative 95% confidence interval (CI) of first new clinical event (AIDS/serious non AIDS/hospitalization/death) in the main analysis (a), in the sensitivity analysis (b).

	N	HR	95% CI		p-value	Adj HR*	95% CI		p-value
(**a) Main analysis** **OUTCOME: time to first new clinical event (AIDS/serious non AIDS/ hospitalization/death)**									
No gap in care	4248	1.00				1.00			
RIC	1062	1.82	1.54	2.14	< 0.001	1.65	1.39	1.95	< 0.001
No gap in care	4248	1.00				1.00			
RIC with HIV-RNA loads ≤ 200 copies/mL at re-entry	356	1.33	0.95	1.84	0.093	1.35	0.97	1.88	0.078
RIC with HIV-RNA loads > 200 copies/mL at re-entry	431	2.06	1.68	2.54	< 0.001	1.79	1.44	2.22	< 0.001
RIC with HIV-RNA loads unknown at re-entry	275	1.85	1.38	2.48	< 0.001	1.66	1.23	2.23	0.001
**(b) Sensitivity analysis (ignoring clinical events occurring within 3 months from baseline)**									
No gap in care	4248	1.00				1.00			
RIC	1062	1.66	1.40	1.97	< 0.001	1.49	1.24	1.77	< 0.001
No gap in care	4248	1.00							
RIC with HIV-RNA loads ≤ 200 copies/mL at re-entry	356	1.20	0.84	1.71	0.324	1.21	0.84	1.74	0.300
RIC with HIV-RNA loads > 200 copies/mL at re-entry	431	1.89	1.52	2.34	< 0.001	1.61	1.29	2.02	< 0.001
RIC with HIV-RNA loads unknown at re-entry	275	1.67	1.22	2.27	0.001	1.49	1.09	2.04	0.013
**(c) Secondary analysis** **OUTCOME: time to first new AIDS event/ AIDS-related death)**									
No gap in care	4248	1.00				1.00			
RIC with HIV-RNA loads ≤ 200 copies/mL at re-entry	356	0.85	0.31	2.33	0.756	0.82	0.30	2.27	0.703
RIC with HIV-RNA loads > 200 copies/mL at re-entry	431	3.91	2.59	5.89	< 0.001	2.70	1.76	4.15	< 0.001
RIC with HIV-RNA loads unknown at re-entry	275	3.67	2.15	6.27	< 0.001	2.79	1.60	4.83	< 0.001
**(d) Secondary analysis** **OUTCOME: time to first new serious non-AIDS event/non-AIDS-related death)**									
No gap in care	4248	1.00				1.00			
RIC with HIV-RNA loads ≤ 200 copies/mL at re-entry	356	1.67	1.08	2.58	0.022	1.72	1.10	2.67	0.017
RIC with HIV-RNA loads > 200 copies/mL at re-entry	431	1.70	1.24	2.32	0.001	1.56	1.13	2.17	0.008
RIC with HIV-RNA loads unknown at re-entry	275	1.52	0.97	2.40	0.070	1.33	0.84	2.10	0.229

Two secondary analyses estimating risk of AIDS event/AIDS-related death (c) and risk of serious non-AIDS event/non-AIDS-related death (d).

*Models were adjusted for: gender, risk factor for HIV transmission, Italian nationality, employment status and level of education, and for the following covariates measured at last follow-up before gap in care: HCV-Ab result, CDC C stage, CD4 count and HIV-RNA, presence of psychiatric co-morbidity and alcohol and/or drug abuse.

When restricting the definition of the outcome to AIDS-related events/death due to AIDS alone, patients who were RIC with HIV-RNA loads > 200 copies/mL had a more than twofold higher risk of developing the endpoint than the unexposed controls (Table 6c). In contrast, patients classified as RIC were at higher risk of serious non-AIDS events or death due to non-AIDS causes than unexposed participants, regardless of the level of HIV-RNA at the time of re-entry (≤ 200 vs. > 200 copies/mL) in the RIC group (Table 6d).

## Discussion

Despite the fact that access to HIV care and treatment is universal and free of charge in Italy, there was a significant proportion of patients who met the definition of LTC in our study.

Indeed, more than one-third of the patients in the Icona Foundation study cohort experienced ≥ 1 gap in care from the start of the observational period, with a reduction in gaps in the most recent years. A similar trend has also been described in a meta-analysis of US studies^10^.

Nevertheless, the proportion of PLWH retained in care in our cohort was substantially higher than that observed in the USA, probably because of the differences in health systems between the two countries leading to different situations in the estimated cascade of care^10,11^. Our study showed that the average time to experiencing the first gap in care was relatively long (13.6 years, 95% CI 13.0–14.3), suggesting that treatment fatigue might appear long after first engaging in care and that physicians treating HIV-positive patients should reinforce adherence to treatment and the need for consistent clinical visits over time. Gaps in care in PLWH have been previously associated with higher mortality^12^.

Regarding the risk factors for poor retention in care, unsurprisingly, our data showed that those who were LTC had a lower socioeconomic status. The characteristics of patients who were LTC were similar to those described in previous studies; for example, in the USA, individuals who were LTC were more frequently African Americans, a population with socio-economic status similar to that of immigrants in Italy^13^.

It is important to note that having a viral load below 200 copies/mL, a proxy for being on cART, was a protective factor against being lost to care, as was previously described in an analysis of the EuroSIDA data^14^. Inconsistent with the results of other studies^15^, alcohol and/or drug abuse and psychological comorbidities were not found to be associated with the risk of becoming LTC in our analysis.

Reassuringly, the incidence of having a gap in care has been stable in recent years. Of note, as shown in both resource-rich and resource-limited countries, among PLWH at high risk of experiencing such gaps, rapid or same-day cART initiation leads to more favourable outcomes^16^ and should be recommended^17^. Nevertheless, the heterogeneity of patients who are typically LTC requires personalized interventions focused on more vulnerable groups, including people who are sceptical of the efficacy of cART^18^. Of note, the majority of person-years of follow-up included in this analysis occurred before the date on which ART initiation regardless of the CD4 count was recommended in the HIV treatment guidelines^17,19–22^. Indeed, Italy usually follows the USA guidelines concerning when to start: the national recommendations for starting cART were a CD4 count < 500 cells/µL from June 1998 to 2000, < 350 CD4/µL from 2001 until 2008, < 500 CD4/µL from 2009 until 2012, and then any count from 2012 onwards.

As anticipated, retention in care is a dynamic process. In the ICONA Foundation Study cohort, 27% of the patients who were LTC re-entered care after a mean gap of 2.7 years; 15% died, and 58% were still classified as lost to follow-up at the time of the analysis, possibly having been transferred to another centre outside of the ICONA Network; having moved abroad, as frequently occurs with immigrants, or having died unrecorded.

We cannot rule out that the underestimation of mortality in this group, given that deaths are reported by the treating physicians with no linkage to the regional or national mortality registry. Interestingly, we found that older participants and those with a lower CD4 count at enrolment in the cohort had a reduced probability of re-entering care.

Approximatively half of the population classified as RIC had HIV-RNA loads ≤ 200 copies/mL at the time of re-entry, suggesting that cART was not interrupted and that this group had only missed blood tests and clinical visits but not treatment. The fact that the CD4 count increased on average during the LTC period supports the hypothesis that ART was never stopped in these patients. Mugavero et al. have previously shown that missing a visit was a risk factor for mortality in the USA^4^. In Italy, patients may continue to receive HIV drugs regardless of whether they attend regular medical visits or undergo blood tests, which is different from the situation in the USA. Nevertheless, despite the observed increase in the CD4 count during the gap, which seemed to have protected these patients from the risk of developing AIDS, we still found evidence of a higher risk of serious non-AIDS events in this group. These results are in agreement with those of a recent study conducted in a cohort of PLWH in Ontario, which showed that the mortality risk and the frequency of use of health care resources were higher among those who were lost to follow-up than among participants who were retained in care^12^.

In contrast, people classified as RIC who had HIV-RNA loads > 200 copies/mL at the time of re-entry into care as a consequence of a decrease in the CD4 count during the gap in care had a higher risk of clinical progression, including new AIDS events. On average, the CD4 count decreased by 100 cells/µL during the gap, and the extent of the decrease was proportional to the length of the gap.

After re-entry into care, patient management was frequently clinically challenging, with participants often presenting with difficult-to-treat single or even multiple opportunistic infections, which are associated with a poor prognosis.

Our analysis showed convincing evidence that people classified as RIC had higher risks of AIDS and non-AIDS events than controls, and this was confirmed in a number of sensitivity analyses. Our data also suggest that the negative impact of experiencing a gap in care may still be present years after returning to care, even after re-starting cART. These results are consistent with those of other previous reports. Indeed, detectable HIV-RNA during the gap was shown to have a potential impact on both the individual level with regard to prognoses and at the population level with regard to increasing the risk of HIV transmission^23,24^. In particular, concerning patient outcomes, cumulative exposure to a high viral load has been previously found to be associated with an increased risk of non-AIDS events, such as myocardial infarction and cancers, such as lymphoma^25–27^.

Moreover, patients who re-started cART after a gap had slower immune reconstitution than that seen after the first initiation of cART, particularly in those older than 40 years^28,29^.

Our study has some limitations. The observational nature of the study design means that residual confounding cannot be ruled out and that there could be bias in in the comparison of patients who were LTC/RIC with controls. In particular, we have shown that immigrants appeared to be at higher risk of becoming LTC after controlling for a number of potential measured confounders, such as the level of education and type of employment. We cannot rule out the presence of residual confounding due to differences in socio-economic status between foreign-born individuals and Italian individuals that are not fully captured by these variables. Second, our study population was a selected group of people who survived the gap in care and are unlikely to have experienced large drops in their CD4 counts; therefore, it is likely that the risk of developing the outcomes has been underestimated. Additionally, because the analysis was conditioned on events that could occur in the future, we cannot rule out that collider bias might have occurred. Moreover, the incidence of mortality could have been underestimated because Icona data are not linked to regional or national mortality registries. Finally, we used a single definition of LTC, regardless of the HIV-RNA load and CD4 count (which may vary by clinical site), patients’ current values of these markers and the time period under observation. However, the results were similar when LTC was defined as an 18-month gap in the main analysis or 12 months in a sensitivity analysis. It was beyond the aim of this analysis to explore strategies to increase retention in HIV care or to evaluate the potential effects of such strategies.

In conclusion, we report precise estimates of the rate of becoming LTC in a large unselected population of PLWH with access to care in Italy over the period from 1997 to 2017 with a median follow-up period of 5 (2.4–8.8) years. Re-entry into care after a period of > 18 months of being LTC appears to be associated with a higher risk of clinical progression regardless of the HIV-RNA load at the time of re-entry into care. These data emphasize the importance of retention in care with regard to reducing the risk of morbidity and mortality in PLWH. This is particularly important in recent times when HIV care has been disrupted by the COVID-19 pandemic. Our analysis also identified subsets of individuals who are at greater risk of morbidity and mortality if they are lost to care, and these individuals should be prioritized when retention efforts are made. Even if early treatment initiation has decreased the proportion of patients disengaging from care, new strategies should be investigated to obtain higher rates of long-term retention in care, especially for the most vulnerable patients.

## Methods

### Study cohort

The ICONA Foundation study is a multicentre prospective observational study of HIV-1-infected patients, which was established in 1997, involving 52 centres for the treatment of infectious diseases across Italy. Enrolled patients were naive to antiretrovirals, regardless of their disease stage and reason for lack of treatment at the time of inclusion in the study. The Icona Foundation study protocol and the related informed consent protocol were approved by the local Ethics Committees of each participating institution (Azienda Ospedaliero-Universitaria Ospedali Riuniti Umberto I-Salesi-Lancisi-Università Politecnica delle Marche, Ancona; Azienda Universitaria Ospedaliera Consorziale—Policlinico di Bari—Ospedale “Giovanni XXIII”, Bari; ASST “Papa Giovanni XXIII”, Bergamo; Azienda Ospedaliero-Universitaria Policlinico di S. Orsola, Università degli Studi di Bologna, Bologna; ASST Spedali Civili-Presidio Ospedaliero di Brescia, Brescia; Azienda Ospedaliera—Ospedale di Circolo—ASST Valle Olona, Busto Arsizio; Azienda Ospedaliero Universitaria di Cagliari—Presidio Ospedaliero Duilio Casula, Cagliari; ARNAS—Presidio Ospedaliero “Garibaldi”—Nesima, Catania; Ospedale “Ss. Annunziata” ASL2 Lanciano Vasto Chieti, Chieti; Azienda Ospedaliera Istituti Ospitalieri, Cremona; Azienda Ospedaliero-Universitaria di Ferrara-Arcispedale Sant’Anna, Ferrara; Ospedale “Santa Maria Annunziata”, Firenze; Ospedale Policlinico “San Martino”—Università di Genova, Genova; Ente Ospedaliero Ospedali Galliera, Genova; Ospedale Santa Maria Goretti, Latina; ASST di Lecco, Ospedale “A. Manzoni”, Lecco; Ospedale Generale Provinciale, Macerata; Azienda Ospedaliera Universitaria Policlinico “G. Martino”, Messina; IRCCS Ospedale San Raffaele Università Vita—Salute, Milano; ASST Fatebenefratelli Sacco—Ospedale Luigi Sacco, Università degli Studi di Milano, Milano; ASST Santi Paolo e Carlo, Università degli Studi di Milano, Milano; Fondazione IRCCS Ca’ Granda Ospedale Maggiore Policlinico, Milano; ASST Grande Ospedale Metropolitano Niguarda, Milano; Azienda Ospedaliero-Universitaria Policlinico di Modena, Modena; ASST di Monza-Ospedale “San Gerardo”, Monza; Azienda Ospedaliera Universitaria “Federico II”, Napoli; Presidio Ospedaliero “D. Cotugno”, Napoli; Azienda Ospedaliera di Padova, Padova; Azienda Ospedaliera Universitaria Policlinico “P. Giaccone”, Palermo; Università degli Studi di Perugia-Ospedale Santa Maria della Misericordia, Perugia; Ospedale Civile Santo Spirito, Pescara; Azienda USL Toscana Centro, Pistoia; IRCCS Arcispedale Santa Maria Nuova, Reggio Emilia; Fondazione Policlinico Universitaria “Agostino Gemelli”—IRCCS Università Cattolica del Sacro Cuore, Roma; IRCCS Istituto Nazionale per le Malattie Infettive Lazzaro Spallanzani, Roma; Azienda Ospedaliero-Universitaria Policlinico Umberto I—Università “La Sapienza”, Roma; Policlinico Tor Vergata—Università degli Studi di Roma “Tor Vergata”, Roma; IFO—Istituto Dermatologico San Gallicano—IRCCS, Roma; Ospedale “Santa Maria della Misericordia”, Rovigo; Azienda Ospedaliero Universitaria di Sassari, Sassari; Azienda Ospedaliero—Universitaria Senese, Siena; Azienda Sanitaria Provinciale 8 di Siracusa—Ospedale “Umberto I”, Siracusa; Azienda Ospedaliera “Santa Maria”, Terni; Ospedale “Amedeo di Savoia”—Università degli Studi di Torino, Torino; Azienda Sanitaria Universitaria Integrata di Udine—Presidio “Santa Maria della Misericordia”, Udine; Ospedale “San Bortolo”—AULSS 8 Berica, Vicenza; and ASL Viterbo Ospedale “Belcolle”, Viterbo).

Written informed consent was obtained from all patients enrolled in the study. All procedures of the study were performed in accordance with the 1964 Helsinki declaration and its later amendments.

For all participants, demographic, clinical and laboratory data (e.g., HIV-RNA load, CD4 count, CD8 count, etc.) and information on treatment were collected prospectively at clinical sites at least every 6 months and recorded online (www.icona.org). Moreover, standard clinical visits are recorded on average every 6 months.

### Participants and definitions

In this study, we included all patients from the ICONA Foundation study database enrolled between January 1997 and March 2017 at 48 of the 52 centres that were still actively recruiting new patients and regularly updating patient follow-up at the time of data extraction. In addition, to be included, participants had to have at least 18 months of follow-up and at least two clinical visits recorded in the database that were separated by ≥ 90 days.

In this analysis, we used the following key definitions:

1. Patients in the cohort who ever experienced a follow-up period of at least 18 months with no recorded clinical visits were defined as ‘lost to care’ (LTC).
2. The subset of LTC patients who, after a gap in care of > 18 months, were subsequently re-engaged in care and then followed-up for at least 1 additional clinical visit were defined as re-engaged in care (RIC).

In the RIC population, the duration of the gap in care was measured as the time between the date of re-entry into care and the last visit prior to the ≥ 18-month gap in care. A person could contribute only his/her first gap to this analysis; subsequent gaps experienced by people who returned to care after this episode were ignored. Participants who were imprisoned, institutionalized or transferred to other clinical centres did not contribute to the gaps in care in this analysis, as it was assumed that they were still receiving care. A detectable viral load at the time of re-entry into care was defined as an HIV-RNA load greater than 200 copies/mL.

### Statistical analysis

#### Predictors of becoming lost to care (LTC) and re-engagement in care (RIC)

The Icona database was frozen for analysis in September 2018. To estimate the incidence of becoming LTC in the cohort, patients’ follow-up was calculated from the date of enrolment in the cohort to the date of the last visit prior to the gap in care (regardless of whether they later re-entered care or not, LTC events) or to the last clinical visit in those retained in case (censored). Incidence rates of becoming LTC per calendar year of observation were estimated. These were calculated as the number of individuals lost to care divided by the PYFU in that year and expressed as rates per 100 PYFU, with 95% confidence intervals (CIs).

In the RIC population, the CD4 count and HIV-RNA load measured at the beginning and the end of the gap were considered, and mean changes were compared with paired Student’s t-tests.

A Cox regression model was used to identify the factors independently associated with the risk of becoming LTC, stratified by clinical centre. The socio-demographic covariates included in the multivariable model were sex, age, nationality (a patient born outside of Italy was considered an immigrant), education level, employment status and route of HIV infection. The clinical covariates included presentation with AIDS, HCV co-infection, HIV-RNA load, CD4 count and calendar year at enrolment. All variables were included in the models as time-fixed covariates measured at enrolment. The role of the time-varying HIV-RNA load on the risk of becoming LTC was also separately investigated using a weighting marginal Cox regression model adjusted for nationality, age at enrolment, HIV risk factors and cART initiation. In a separate Cox model, we evaluated an alternative endpoint after excluding LTC patients who subsequently re-engaged in care.

In the LTC group, a logistic regression model was used to identify factors associated with re-entry into care in the subgroup with an HIV-RNA load > 200 copies/mL compared to those who never re-entered care.

All models included all the covariates listed above, selected a priori as potential confounders on the basis of associations previously shown in the literature or axiomatic knowledge, and all models were also adjusted for calendar year of enrolment.

#### Clinical progression

In the second part of the analysis, we focused on the possible role of a gap in care longer than 18 months with regard to modifying the risk of clinical progression once the person had re-entered care. This question was addressed by comparing the RIC (exposed) population with a control group of unexposed patients who were continuously retained in case. The baseline for the analysis was the date of re-entry into care for cases and the corresponding index date for controls. The index date for controls was after a time from entry that matched the length of time from entry to re-engagement in care of the corresponding patient who became RIC. Two additional matching variables were considered: age [< 30, 30–40, 40–50, > 50 years] and calendar year at enrolment [1997–1998, 1999–2001, 2002–2004, 2005–2007, 2008–2010, 2011–2013, 2014–2017]. Each control could be matched to one or more cases to achieve a ratio of 1:4 between exposed and unexposed individuals.

For each participant, follow-up accrued from baseline to the date of clinical progression/last follow-up visit.

Clinical progression was the composite endpoint defined at the time at which a participant first experienced one of the following events:

- death due to any cause;
- new occurrence of AIDS-related opportunistic infection or neoplasm;
- new occurrence of serious non-AIDS-related event; or
- new occurrence of hospitalization.

AIDS-related opportunistic infections and neoplasms were defined according to the Centre for Disease Control and Prevention 1993 classification system.

Serious non-AIDS-related events included the following: any non-AIDS-related malignancy, cerebro-cardio-vascular events (acute myocardial infarction, coronary disease requiring invasive procedures, carotid endarterectomy, stroke, cerebral haemorrhage), end-stage liver disease (decompensated cirrhosis, i.e., spontaneous bacterial peritonitis, variceal bleeding, portosystemic encephalopathy, refractory ascites, hepatic-renal syndrome, HCC) and end-stage renal disease (defined as confirmed estimated glomerular filtrate rate < 30 mL/min or kidney failure requiring dialysis or renal transplantation).

A standard Cox regression model with time-fixed covariates was used to compare the hazard ratio (HR) for experiencing clinical progression in participants with and without gaps in care (RIC population vs matched controls). In a secondary analysis, we divided the RIC population into two groups according to the HIV-RNA load at the time of re-engagement in care (≤ 200 or > 200 copies/mL) and determined the HR for clinical progression by comparing patients with regular follow-up to two groups, namely, the RIC population with HIV-RNA loads ≤ 200 copies/mL and the RIC population with HIV RNA loads > 200 copies/mL at the time of re-engagement in care. Time-fixed covariates included in the multivariable analysis were sex, risk factors for HIV transmission, nationality, employment status and level of education. Again, potential confounders of the association between becoming RIC and the risk of the clinical outcome that were included in the multivariable model were selected a priori on the basis of associations previously shown in the literature or axiomatic knowledge.

The dataset included repeated measurements for HCV-Ab results, CDC C stage, CD4 count and HIV-RNA load, any psychiatric comorbidities and alcohol or drug abuse. All these variables were included in the Cox regression model and included as time-fixed covariates as the value that was recorded at the last visit prior to the gap in care for the RIC group and at on the date of matching for the control group. Of note, this is not the same date that was previously referred to as the ‘index date’, which is the date in the unexposed group matching the date of re-entry into care in the RIC group. This was done because the values measured at that index date were likely to be a consequence of experiencing the gap in care and not a possible cause and were therefore likely to be mediators rather than potential confounding factors.

The proportional hazards assumption was verified by testing the interaction between each of the covariates and the natural logarithm of survival time. All models were stratified by clinical centre.

A sensitivity analysis was performed in which LTC was defined by a gap of > 12 months instead of 18 months. Additionally, as we speculated that the clinical development of symptoms could have been the cause of return to care for many patients, we conducted a sensitivity analysis after ignoring clinical events occurring in the first 3 months after the date of re-engagement in care. Moreover, two further sensitivity analyses were conducted: one counted only AIDS events or deaths due to AIDS and one counted only serious non-AIDS events or non-AIDS-related deaths as outcome.

STATA software (version 15.1) was used for all analyses.

## Supplementary Information

Supplementary Information.

## References

[1] Croxford S, Kitching A, Desai S (2017). Mortality and causes of death in people diagnosed with HIV in the era of highly active antiretroviral therapy compared with the general population: An analysis of a national observational cohort. Lancet Public Health.

[2] Accessed 01 June 2020. Data available at https://www.epicentro.iss.it/aids/notiziario-coa.

[3] Giordano TP, Gifford AL, White AC (2017). Retention in care: A challenge to survival with HIV infection. Clin. Infect. Dis..

[4] Mugavero MJ, Lin HY, Willig JH (2009). Missed visits and mortality among patients establishing initial outpatient HIV treatment. Clin. Infect. Dis..

[5] Yehia BR, French B, Fleishman JA (2014). Retention in care is more strongly associated with viral suppression in HIV-infected patients with lower versus higher CD4 counts. J. Acquir. Immune Defic. Syndr..

[6] Gardner EM, McLees MP, Steiner JF (2011). The spectrum of engagement in HIV care and its relevance to test-and-treat strategies for prevention of HIV infection. Clin. Infect. Dis..

[7] Scourfield A, Jacksonn A, Nelson M (2011). Will earlier diagnosis of HIV infection in late presenters reduce the frequency of serious opportunistic infections?. HIV Med..

[8] Lee MJ, Rayment M, Scourfield A, Gazzard B (2013). Comparison of two cohorts of patients presenting with AIDS: Patients with previously known HIV diagnoses and true late presenters. Sex. Transm. Infect..

[9] Colasanti J, Stahl N, Farber E, Del Rio C, Armstrong W (2017). An exploratory study to assess individual and structural level barriers associated with poor retention and re-engagement in care among persons living with HIV/AIDS. J. Acquir. Immune Defic. Syndr..

[10] Marks G, Gardner LI, Craw J, Crepaz N (2010). Entry and retention in medical care among HIV-diagnosed persons: A meta-analysis. AIDS.

[11] Rebeiro PF, Gange SJ, Horberg MA (2016). Geographic variations in retention in care among HIV-infected adults in the United States. PLoS ONE.

[12] Kendall CE, Raboud J, Donelle J (2019). Lost but not forgotten: A population-based study of mortality and care trajectories among people living with HIV who are lost to follow-up in Ontario, Canada. HIV Med..

[13] Gourlay A, Noori T, Pharris A (2017). The human immunodeficiency virus continuum of care in European Union Countries in 2013: Data And challenges. Clin. Infect. Dis..

[14] Mocroft A, Kirk O, Aldins P (2008). Loss to follow-up in an international, multicentre observational study. HIV Med..

[15] De Boni RB, Peratikos MB, Shepherd BE (2018). Is substance use associated with HIV cascade outcomes in Latin America?. PLoS ONE.

[16] Labhardt ND, Ringera I, Lejone TI (2018). Effect of offering same-day ART vs usual health facility referral during home-based HIV testing on linkage to care and viral suppression among adults with HIV in Lesotho The CASCADE randomized clinical trial. JAMA.

[17] Panel on Antiretroviral Guidelines for Adults and Adolescents. *Guidelines for the Use of Antiretroviral Agents in Adults and Adolescents with HIV. Department of Health and Human Services* (2019). Accessed 01 June 2020. https://clinicalinfo.hiv.gov/sites/default/files/inline-files/AdultandAdolescentGL.pdf.

[18] Shubber Z, Mills EJ, Nacheda JB (2016). Patient-reported barriers to adherence to antiretroviral therapy: A systematic review and meta-analysis. PLoS Med..

[19] EACS Guidelines, version 10.0 (2019). Accessed 01 June 2020. https://www.eacsociety.org/files/2019_guidelines-10.0_final.pdf.

[20] Saag MS, Benson CA, Gandhi RT (2018). Antiretroviral drugs for treatment and prevention of HIV infection in adults: 2018 recommendations of the International Antiviral Society-USA Panel. JAMA.

[21] Update of recommendations on first- and second-line antiretroviral regimens (2019). https://www.who.int/hiv/pub/arv/arv-update-2019-policy/en/.

[22] BHIVA guidelines for the treatment of HIV-1-positive adults with antiretroviral therapy 2015 (2016 interim update) (2016). Accessed 01 June 2020. https://www.bhiva.org/HIV-1-treatment-guidelines.

[23] Cohen MS, Chen YQ, McCauley M, Gamble T, Hosseinipour MC, Kumarasamy N (2016). Antiretroviral therapy for the prevention of HIV-1 transmission. N. Engl. J. Med..

[24] Rodger AJ, Cambiano V, Bruun T (2019). Risk of HIV transmission through condomless sex in serodifferent gay couples with the HIV-positive partner taking suppressive antiretroviral therapy (PARTNER): Final results of a multicentre, prospective, observational study. Lancet.

[25] Delaney JA, Nance RM, Whitney BM (2019). Cumulative human immunodeficiency viremia, antiretroviral therapy, and incident myocardial infarction. Epidemiology.

[26] Park LS, Tate JP, Sigel K (2018). Association of viral suppression with lower AIDS-defining and non-AIDS-defining cancer incidence in HIV-infected veterans: A prospective cohort study. Ann. Intern. Med..

[27] Zoufaly A, Stellbrink HJ, Heiden MA (2009). Cumulative HIV viremia during highly active antiretroviral therapy is a strong predictor of AIDS-related lymphoma. J. Infect. Dis..

[28] Touloumi G, Pantazis N, Stirnadel HA (2008). Rates and determinants of virologic and immunological response to HAART resumption after treatment interruption in HIV-1 clinical practice. J. Acquir. Immune Defic. Syndr..

[29] Mussini C, Touloumi G, Bakoyannis G (2009). Magnitude and determinants of CD4 recovery after HAART resumption after 1 cycle of treatment interruption. J. Acquir. Immune Defic. Syndr..

