# Supplementary material for: Determinants of loss to care and risk of clinical progression in PLWH who are re-engaged in care after a temporary loss
Source: Sci Rep. 2021 May 5;11:9632. doi: 10.1038/s41598-021-88367-5 (PMC8099893; doi:10.1038/s41598-021-88367-5)
Supplement: Supplementary file 1 — Supplementary Information. [file 41598_2021_88367_MOESM1_ESM.docx]

**Determinants of loss to care and risk of clinical progression in PLWH who are re-engaged in care after a temporary loss**

Cristina Mussini ^1^, Patrizia Lorenzini ^2*^, Alessandro Cozzi Lepri ^3^, Alessia Mammone ^2^, Giovanni Guaraldi ^1^, Giulia Marchetti ^4^, Miriam Lichtner ^5^, Giuseppe Lapadula ^6^, Sergio Lo Caputo^7^, Andrea Antinori ^2^, Antonella d’Arminio Monforte ^4^, Enrico Girardi ^2^

^1^Infectious Diseases Clinic, University of Modena and Reggio Emilia, Modena, Italy

^2^National Institute for Infectious Diseases ‘L. Spallanzani’, Rome, Italy

^3^ Institute for Global Health, UCL, London, UK

^4^Clinic of Infectious Diseases, San Paolo Hospital, University of Milan, Milan, Italy

^5^Department of Public Health and Infectious Diseases Unit, Sapienza University of Rome, Polo Pontino, Latina, Italy

^6^Division of Infectious Diseases, San Gerardo Hospital, Monza, Italy

^7^Clinic of Infectious Diseases, University of Foggia, Foggia, Italy

| **Sensitivity analysis-2** | **N** | **HR** | **95%CI** | | **p-value** | **Adj HR*** | **95%CI** | | **p-value** |
| --- | --- | --- | --- | --- | --- | --- | --- | --- | --- |
| **No gap in care** | 10,603 | 1·00 | ·· | ·· | ·· | 1·00 | ·· | ·· | ·· |
| **RIC** | 2,763 | 1·37 | 1·23 | 1·53 | <0·001 | 1·20 | 1·07 | 1·35 | 0·002 |
| **No gap in care** | 10,603 | 1·00 | ·· | ·· | ·· |  | ·· | ·· | ·· |
| **RIC & HIV-RNA<200 copies/mL at re-entry** | 1,630 | 0·96 | 0·81 | 1·14 | 0·619 | 0·97 | 0·81 | 1·15 | 0·690 |
| **RIC & HIV-RNA>200 copies/mL at re-entry** | 988 | 1·79 | 1·56 | 2·05 | <0·001 | 1·40 | 1·21 | 1·62 | <0·001 |
| **RIC & HIV-RNA unknown at re-entry** | 118 | 2·61 | 1·39 | 4·87 | 0·003 | 2·05 | 1·10 | 3·85 | 0·025 |

**Supplementary table 1. Crude and adjusted Hazard Ratio (adj HR) and 95%CI of first new clinical event (AIDS/serious non AIDS/hospitalization/death). Gap in care was defined as no clinical visit for at least 12 months.**

*****Models were adjusted for: gender, risk factor for HIV transmission, Italian nationality, employment status and level of education, and for the following covariates measured at last follow-up before gap in care: HCV-Ab result, CDC C stage, CD4 count and HIV-RNA, presence of psychiatric co-morbidity and alcohol and/or drug abuse
